# Carotid intima-media thickness is associated with cognitive deficiency in hypertensive patients with elevated central systolic blood pressure

**DOI:** 10.1186/1476-7120-10-41

**Published:** 2012-10-18

**Authors:** Eros da Mota Dias, Luiz Tadeu Giollo, Débora Dada Martinelli, Camila Mazeti, Heitor Moreno Júnior, José Fernando Vilela-Martin, Juan Carlos Yugar-Toledo

**Affiliations:** 1 Hypertension Clinic, Department of Internal Medicine, State Medical School of São José do Rio Preto (FAMERP), Rua: Las Vegas 200, São José do Rio Preto, SP CEP 15093-010, Brazil; 2 Cardiovascular Pharmacology Laboratory, Faculty of Medical Sciences, State University of Campinas (UNICAMP), Campinas, SP CEP 13414-093, Brazil

**Keywords:** Hypertension, Cognitive impairment, Intima-media thickness, Central systolic blood pressure

## Abstract

**Background:**

The role of hypertension in the loss of cognitive function is controversial. Relationships between hypertension and increases in cerebral vascular resistance, diffused lesions and multiple lacunar infarcts of the white matter are well known. Thus, the objectives of this study were: to evaluate the relationship between hypertension and cognitive dysfunction (CD), identify risk factors and determine the association between early markers of vascular disease and CD in hypertensive individuals.

**Methods:**

Two hundred individuals aged between 40 and 80 years old were evaluated in this cross-sectional prospective study. Fifty participants were controls (CT). The remaining 150 hypertensive patients were subdivided into two groups, those with CD (HCD) and those without CD (HNCD). All participants underwent clinical evaluations and biochemical blood tests were performed. CD was investigated using the Mini Mental State Examination (MMSE) following the guidelines for its use in Brazil. The impact of hypertension on the arterial bed was assessed by identifying and measuring changes in the intima-media thickness (IMT) by vascular ultrasonography of the carotid arteries and analyses of the central blood pressure and Augmentation Index by applanation tonometry of the radial artery.

**Results:**

There were no significant differences in the total cholesterol, high-density lipoprotein cholesterol and triglycerides plasma concentrations between the three groups. The serum creatinine and estimated glomerular filtration rate were within normal ranges for all three groups. A significantly lower MMSE score was recorded for the HCD Group compared to the HNCD and CT Groups (p-value < 0.05).

The IMT was significantly different between the HNCD and HCD Groups (p-value = 0.0124). A significant difference in the IMT was also observed between hypertensive patients and the CT Group (p-value < 0.0001). Age, low-density cholesterol, high-density cholesterol, triglycerides and IMT increased the Odds Ratio for cognitive dysfunction.

The central systolic pressure was significantly higher in the HCD and HNCD Groups compared to CT Group (p-value < 0.0001).

**Conclusions:**

Hypertensive patients with CD have changes in the vascular morphology characterized by an increased carotid IMT, enhanced atherosclerotic lipid profile and impaired hemodynamic functional manifested by elevated central systolic blood pressure.

## Introduction

Hypertension is a major risk factor for cardiovascular disease including strokes (especially hemorrhagic), coronary heart disease (myocardial infarction) [1-3], left ventricular hypertrophy, congestive heart failure [4-6], aortic dissection [7-9], renal failure [10-12] and peripheral vascular disease [13]. Acute myocardial infarction and strokes are associated with high mortality rates in adulthood, particularly in the elderly [14-16].

Cognitive deficiency (CD) is considered a form of dementia and defined as a “persistent or permanent decline in varying dimensions of intellectual function in a way that it interferes with the individual’s normal social or economic activities. Currently, the Diagnostic and Statistical Manual of Mental Disorders defines dementia as an ‘organic brain syndrome’ (American Psychiatric Association, 1994); this is mainly to differentiate it from other cognitive function disorders such as mental retardation and reversible episodes of amnesia or other changes in focal elements of cognitive components such as language disorders[17,18].

The role of hypertension in determining the loss of cognitive function is not well defined [19,20]. It is well known that hypertension is associated to increased cerebral vascular resistance with diffuse lesions and multiple lacunar infarcts in the white matter (especially in the subcortical region) that are histopathologically detectable and visible by magnetic resonance imaging [21-23]. Such infarcts have been linked to dementia in the elderly [24]. With the current scientific and technological progress, more accurate diagnostic tests such as positron emission tomography allow a better understanding of changes in human brain structures related to hypertension, as do animal experiments, which allow a histopathological view of similar lesions [25-29]. Thus, vascular risk factors may impair cognitive function and are linked not only to vascular dementia but also to Alzheimer’s disease. The level of evidence for these associations is highest for hypertension and diabetes mellitus, especially when these factors are found in middle-aged individuals [30].

The effect of antihypertensive treatment on the cognitive function in older adults is also controversial. Some studies suggest that treatment improves cognitive function while others found no effect or even the opposite effect [31-34]. In general, research has difficulty in distinguishing the true influence of hypertension on cognition as the effect of antihypertensive medications must be taken into account [35-38]. This is because of several reasons; in general, adequate blood pressure (BP) control is not achieved with the treatment regimens both due to non-adherence to treatment and due to the inappropriate choice of antihypertensive medications. It is well known that hypertension should be treated with drug combinations when the desired effect of a drop in BP is not attained with a single medication [39,40].

Thus, the objectives of this study were to evaluate the relationship between hypertension and CD, to identify risk factors associated to the development of CD (gender, age, education, body mass index (BMI), dyslipidemia, diabetes and chronic kidney disease) and to determine associations between different early markers of vascular disease (carotid intima-media thickness, vascular elasticity and rigidity, and central arterial pressure) and CD in hypertensive individuals on regular antihypertensive treatment.

## Patients and methods

The sample population of this prospective study consisted of two hundred consecutive male and female individuals aged between 40 and 80 years old selected in the specialized hypertension center of the Medicine School in São José do Rio Preto (FAMERP). All participants were submitted to ultrasound assessment and the Mini Mental State Examination between February 2009 and October 2011. Of these, one hundred and fifty were stage II hypertensive patients according to JNC VII [39]; Forty-two hypertensive patients had CD (HCD group) and one hundred and eight hypertensive patients had no CD (HNCD group).

The Control Group (CT), made up of non-hypertensive, non-cognitive dysfunction individuals, was selected from subjects referred to investigate stage I primary hypertension but whose blood pressures proved to be normal after standard measurements in the physical examination and whose biochemical blood tests were normal.

This study was approved by the Research Ethics Committee of the Post Graduation Program in Health Sciences of the Medicine School in São José do Rio Preto (FAMERP). Informed written consent was obtained from all participants before their enrollment in the study. The sample size was estimated based on an 80% statistical power to demonstrate the objectives of the study with a type I alpha error rate of 5%.

The inclusion criteria of the hypertensive patients were: age between 40 and 80 years under treatment for stage II hypertension - VII JNC, optimal response to pharmacological treatment (BP < 140/90 mmHg) with no acute medical illness in the previous month and the ability to understand, verbalize and answer questions related to the study. The exclusion criteria were the presence of atherosclerotic plaque defined as a focal structure that encroaches into the arterial lumen by at least 0.5 mm or 50% of the surrounding IMT or demonstrates a thickness > 1.5 mm as measured from the media-adventitia interface to the intima-lumen interface.

Participants were submitted to clinical evaluations, physical examinations and investigations of their family history for cardiovascular disease. Blood pressure of the right arm was measured during the morning by a trained healthcare professional with the patient in the seated position and using an appropriately sized cuff.

The age, gender, weight, height, and the results of an electrocardiogram and biochemical blood tests were recorded. Patients with secondary hypertension, those taking anticholinesterase drugs, those that had suffered strokes, had diagnosis of dementia or had recently had an acute infection were excluded in order to rule out the possibility of acute confusional state. Additionally, patients with family history of Alzheimer’s disease (parents and siblings), or history of psychiatric illness or substance abuse were excluded.

## Mini-mental state examination

The Mini-Mental State Examination (MMSE) is a test that uses two specific types of responses, verbal and nonverbal, to identify cases of CD. Diagnosis of dementia or any specific dementing illness is not an objective of this test. Responses to questions are studied through verbal sub-tests that measure time-space orientation, immediate memory, attention and calculation, and recall and language and nonverbal subtests that assess perceptual-motor coordination and comprehension of instructions. The test quantifies responses through simple, easy to use criteria that are compatible with the application. Administration of the test takes about 5 to 10 minutes by duly trained professionals. The test is dependent on the level of education of the interviewee. The MMSE results in a maximum score of 30 points where the lower the score, the greater the CD with 24 points originally being considered as the cutoff point [41-46].

## Blood pressure measurements

The starting point to evaluate hypertension is the accuracy of the BP measurement. The average of three measurements of the systolic blood pressure (SBP) and diastolic blood pressure (DBP) were recorded. Measurements were made by the indirect technique using OMRON equipment and employing cuffs with appropriate dimensions for the perimeter of the arm (a width-length ratio of 1:2). The readings were taken after the patient had remained resting in the seated position with the arm on a support at the same height as the apex in a quiet environment for 5 to 15 minutes, at least 60 minutes after the ingestion of coffee and 30 minutes after smoking. BP analyses were performed at least three times with an interval of five minutes between readings and the mean of measurements was calculated.

## Carotid intima-media thickness

The carotid intima-media thickness (IMT), the mean thickness of the anterior and posterior walls of the left and right carotid arteries, was evaluated using high resolution ultrasound. Patients were always examined between 7:00 a.m. and 11:00 a.m. in the supine position with the head at a 45° angle on a support.

This method is established and standardized according to the “34^th^ Report of the Bethesda Conference Task Force #3 Noninvasive Atherosclerosis Measurement” [47] using a sample protocol outlined by the American Society of Echocardiography [48].

The ultrasound analysis was performed by a physician experienced in vascular studies and certified by the Cardiovascular Imaging Department of the Brazilian Society of Cardiology and blinded to the patient's clinical data. The examination was carried out according to a previously established protocol [49] standardized for both carotid arteries. Images were acquired at end diastole (defined as the R wave of the electrocardiogram) using a 7–12 MHz linear transducer and high resolution ultra-sound (Philips, HD 11 XE, Andover MA – USA). The intima-media thickness was measured over a 1-cm segment of the artery located approximately 0.5 cm below the carotid-artery bulb and considered not to contain any plaque (i.e., not to have any perceivable protrusion of the artery wall into the lumen) [50,51].

The IMT data were analyzed offline by two independent observers by computer program analysis (M’ATh - Metris, France) which allows measurement of the IMT from images obtained during four cardiac cycles identified by the R wave of the ECG. The analysis, based on the tone of the gray scale and a specific algorithm to recognize tissue, is automatic. The average measurements of the near and far walls of the left and the right common carotid arteries were used to compare the results between the three study groups. The variability between IMT measurements should be less than 2%, as in this study.

## Radial artery tonometry and central systolic blood pressure

Central (aortic and carotid) blood pressures are more strongly related to the pathogenesis of cardiovascular disease than peripheral pressures [52]. It is the aortic systolic pressure that the left ventricle encounters during systole (afterload), and the aortic pressure during diastole is a determinant of coronary perfusion. Furthermore, the distending pressure in the large elastic-type arteries (aorta and carotid) is a key determinant of the degenerative changes that characterize accelerated aging and hypertension. In contrast, muscular peripheral arteries, such as the brachial and radial arteries, are less influenced by these changes [53].

Because of pulse pressure amplification between central and peripheral arteries, it is inaccurate to use the brachial pulse pressure as a surrogate for carotid or aortic pulse pressure, particularly in young subjects. The carotid femoral Pulse Wave Velocity (PWV) is considered the “gold standard” measurement of arterial stiffness. Pulse-wave analysis should be obtained at the central level, i.e. at the site of the carotid artery or the ascending aorta, and either directly recorded or computed from the radial artery waveform using a transfer function. The pulse waveform should be analyzed by three major parameters: central pulse pressure, central systolic blood pressure and augmentation index [54].

Arterial stiffness is one of the determinants of increased pulse pressure (PP) and central BP, which are considered predictors of cardiovascular risk.

Tonometry of the radial artery provides an accurate, reproducible, noninvasive assessment of the central PP waveform. Radial artery applanation tonometry (AT) is performed by placing a hand-held tonometer (strain gauge pressure sensor) over the radial artery and applying mild pressure to partially flatten the artery. The radial artery pressure is then transmitted from the vessel to the sensor (strain gauge) and is recorded digitally. A mathematical formula using a fast Fourier transformation algorithm, approved by the Food and Drug Administration of the USA, permits derivation and calculation of central systolic blood pressure indices from a peripheral brachial blood pressure with concomitant recording of a PP wave with radial AT. This provides information on the functional condition of arteries by the Augmentation Index, which calculates the ratio of the reflected wave and the ejection wave [51]. The premise states that the speed at which these waves travel increases as the rigidity of the arterial wall increases. Thus, endothelial disorders with reduced function can be detected early by the AT system [55-58].

The examination to evaluate the AT was made with the patient in a quiet environment, after resting for at least 5 minutes, sitting with the legs uncrossed and the bladder empty, away from acute stressors, without having drunk alcoholic beverages and without having smoked for at least 30 minutes. The OMROM AT equipment with a radial ultrasound transducer was employed with cuffs of suitable dimensions and correct proportion for the arm circumference.

## Statistical analysis

Descriptive statistics were performed to describe the sample in relation to demographic and clinical characteristics. Means, standard deviation and 95% confidence interval were calculated for all variables. Antihypertensive drugs by class (Hydrochlorothiazide, ACE-inhibitors, Angiotensin type 2 receptor blockers, Beta-blockers and Calcium channel blockers) were entered as binary values.

For the analysis of quantitative parameters, the *t*-test and Mann–Whitney test were used to compare aspects of hypertensive patients with or without changes in cognitive function and control individuals. The chi-square test and proportions were adopted for qualitative variables.

Analysis of variance (ANOVA) was used to compare the mean of the carotid intima thickness, central SBP and Augmentation Index between the three groups.

Logistic regression, with the STATA 12.1 statistics program (Stata Corp LP, College Station, Texas, USA), was used to determine if independent variables can predict the outcome, in this case CD (the dependent variable). The independent variables were the clinical and demographic variables including, age, gender, BMI, blood sugar levels, total cholesterol, LDL cholesterol, HDL cholesterol, triglycerides, creatinine, estimated glomerular filtration rate, microalbuminuria, 24-hour urinary sodium excretion, systolic blood pressure, diastolic blood pressure, pulse pressure, mean blood pressure, augmentation index (Ai75), central systolic blood pressure (CSBP) and carotid IMT. Statistical significance was set for a p-value < 0.05.

## Results

There were statistically significant differences between the groups in respect to age (HCD, HNCD vs. CT - p-value < 0.0001; HCD vs. HNCD - p-value = 0.0017) and BMI (HCD vs. CT - p-value < 0.0001; HNCD vs. CT - p-value < 0.0001). No significant differences were found between the two hypertensive subgroups in relation to the duration of hypertension (HCD = 15.03 ± 9.74 vs. HNCD 15.73 ± 9.57 - p-value = 0.40) and in relation to the use of different anti-hypertensive drug classes (Table 1).

**Table 1:** Demographic and anthropometric characteristics of the HCD, HNCD and CT groups

	**HCD**	**HNCD**	**CT**
**Age (years)**	63.22 ± 9.3*	55.75 ± 8.66*	44.56 ± 7.93
**Gender: F/M**	26/16	58/50	30/18
**Height**	1.62 ± 0.11	1.65 ± 0.09	1.63v0.24
**weight**	75.56 ± 15.95	80.69 ± 16.18	71.58v18.25
**BMI (kg/m**^**2**^**)**	29.04 ± 6.81*	29.57 ± 5.2*	25.83 ± 4.91
**Duration HBP**	15.03 ± 9.74	15.73 ± 9.57	-
**Number of A-H**	2.43 ± 0.97	2.35 ± 0.85	
** HCT**	36 (86%)	85 (79%)	
** ACEI**	22 (52%)	53 (49%)	
** ARB**	15 (36%)	45 (42%)	
** BB**	16 (38%)	40 (37%)	
** CCB**	13 (31%)	31 (29%)	
**n**	42	108	48

Values expressed as means ± standard deviation or n (%).

*n* number of individuals, *HCD* hypertensive with cognitive deficiency, *HNCD* hypertensive without cognitive deficiency, *CT* controls, *BMI* body mass index, *M* male, *F* female, *HBP* high blood pressure.

*A-H* Anti-hypertensive drugs, *HCT* Hydrochlorothiazide, *ACEI* Angiotensin Converting Enzyme Inhibitor, *ARB* Angiotensin Type 2 Receptor Blocker, *BB* beta blocker, *CCB* Calcium Chanel Blocker.

* p-value < 0.0001 HCD-HNCD vs. CT.

The mean values of SBP, DBP, MAP and PP in the HCD and HNCD groups measured during outpatient visits were all higher than in the CT group. There were statistically significant differences for the SBP and PP of the HCD and HNCD groups versus the CT group (p-value < 0.001). There was no statistically significant difference for DBP and heart rate (HR) comparing the three groups (Table 2).

**Table 2:** Hemodynamic characteristics of the HCD, HNCD and CT groups

	**HCD**	**HNCD**	**CT**
**SBP (mmHg)**	132.9 ± 24.29*	128.4 ± 16.66**†**	115.4 ± 20.61
**DBP (mmHg)**	73.26 ± 15.24	73.65 ± 13.32	70.49 ± 13.25
**MBP (mmHg)**	93.13 ± 16.61	91.90 ± 13.12	85.44 ± 15.45
**PP (mmHg)**	59.61 ± 18.41*	54.75 ± 13.17**†**	44.96 ± 9.53
**HR (bpm)**	68.00 ± 13.1	71.91 ± 11.29	72.78 ± 11.08
**n**	42	108	48

Values expressed as means ± standard deviation.

*n* number of individuals, *HCD* hypertensive with cognitive deficiency, *HNCD* hypertensive without cognitive deficiency, *CT* controls, *SBP* systolic blood pressure, *DBP* diastolic blood pressure, *MBP* mean blood pressure, *PP* pulse pressure, *HR* heart rate.

* p-value < 0.0001 HCD vs. CT; **†** p-value < 0.002 HNCD vs. CT.

The biochemical values of the three groups are described in Table 3.

**Table 3:** Biochemical parameters of the HCD, HNCD and CT groups

	**HCD**	**HNCD**	**CT**
**Glycemia (mg/dL)**	106.7 ± 23.26	116.9 ± 42.71*	91.04 ± 25.62
**Total cholesterol (mg/dL)**	186.9 ± 36.59	184.6 ± 38.62	201.9 ± 44.82#
**LDL cholesterol (mg/dL)**	102.6 ± 30.71	96.62 ± 28.96	124.4 ± 34.94
**HDL cholesterol (mg/dL)**	57.68 ± 12.59	51.16 ± 13.19	54.28 ± 14.37
**Triglycerides (mg/dL)**	159.5 ± 12.91	153.1 ± 138.2	121.1 ± 58.31
**Creatinine (mg/dL)**	1.08 ± 0.17 †	1.13 ± 0.28 †	0.76 ± 0.21
**eGFR**	70.14 ± 21.77	73.31 ± 22.65	98.16 ± 18.9
**Microalbuminuria mg/24 h**	49.24 ± 72.76	64.60 ± 202.7	8.6 ± 13.8
**Potassium (mEq/L)**	4.27 ± 0.27	4.28 ± 0.42	4.24 ± 0.08
**24 h Urinary Sodium (mEq/24 h)**	206.0 ± 72.83 ≠	204.2 ± 80.82 ≠	169.1 ± 55.23
**n**	41	108	48

Values expressed as means ± standard deviation.

*n* number of individuals, *HCD* hypertensive with cognitive deficiency, *HNCD* hypertensive without cognitive deficiency, *CT* controls, *LDL* low-density lipoprotein cholesterol fraction, *HDL* high-density lipoprotein cholesterol fraction; *eGFR* estimated glomerular filtration rate.

* # † ≠ p-value < 0.0001.

**Figure 1:**
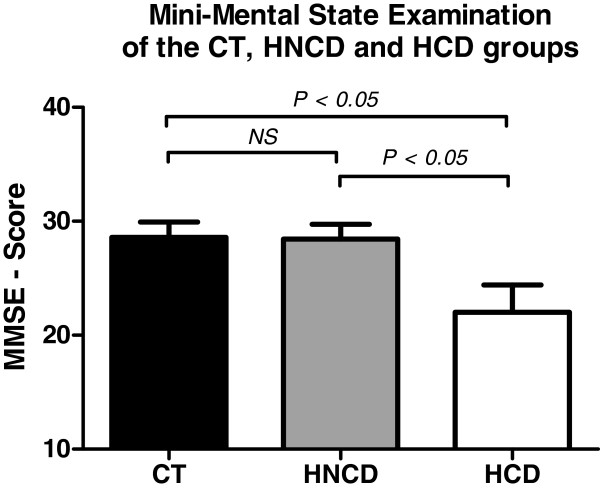
**Assessment score of the Mini-Mental State Examination for the CT, HNCD and HCD groups. **MMSE = Mini-Mental State Examination; CT = controls; HNCD = arterial hypertension without cognitive deficiency; HCD = arterial hypertension with cognitive deficiency.

The results of the MMSE for the three groups (HCD, HNCD and CT) expressed as means ± standard deviation are summarized in Table 4. A significantly lower MMSE score was found for the HCD group compared to the HNCD and CT groups (Figure 1).

**Table 4:** Mini-Mental State Examination Score for the HCD, HNCD and CT groups

**Group**	**Mean**	**SD**	**95% CI**
**CT**	27.77	4.4	26.46 - 28.08
**HNCD**	28.44	1.30	28.19 - 28.69
**HCD**	22.02	2.4	21.27 - 22.78

*n* number of individuals, *HCD* hypertensive with cognitive deficiency, *HNCD* hypertensive without cognitive deficiency, *CT* controls *SD* standard deviation, *95% CI* = 95% confidence interval.

**Figure 2:**
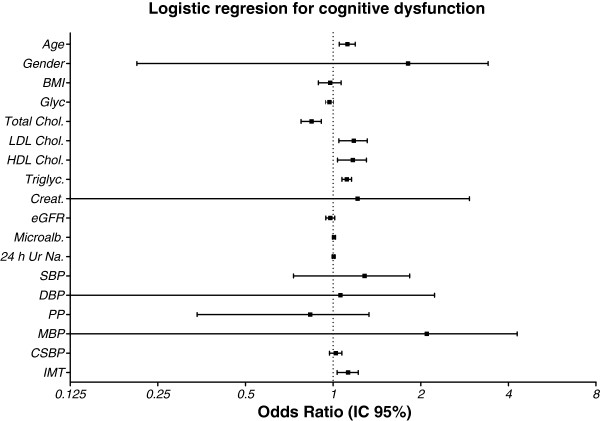
**Logistic regression for event cognitive dysfunction in hypertensive patients. **Odds Ratio (OR) and Confidence Interval 95% (CI 95%). BMI – body mass index; LDL - low-density lipoprotein cholesterol fraction; HDL - high-density lipoprotein cholesterol fraction; eGFR – estimated glomerular filtration rate; SBP = systolic blood pressure; DBP = diastolic blood pressure; PP = pulse pressure; MBP = mean blood pressure; CSBP = central systolic blood pressure; IMT = carotid intima-media thickness.

The logistic regression analysis of risk factors for predictive effect on CD in hypertensive patients (age, gender, height, weight, BMI, blood sugar levels, total cholesterol, HDL cholesterol, LDL cholesterol, triglycerides, creatinine, estimated glomerular filtration rate, microalbuminuria, and 24-hour urinary sodium excretion) showed that only age HDL cholesterol, LDL cholesterol, triglycerides and increased IMT were associated with increased odds ratios (OR) in hypertensive patients with declines in MMSE scores (Table 5 and Figure 2).

**Table 5:** Logistic regression, Odds Ratio (OR) and 95% Confidence Interval (CI) for cognitive dysfunction in relation to cardiovascular risk factors

	**Odds Ratio**	**SD**	**p-value**	**95% CI**	
**Age**	1.113	0.406	0.048*	1.043	1.181
**Gender**	1.339	0.673	0.562	0.499	3.587
**BMI**	0.975	0.044	0.580	0.891	1.066
**Glycemia**	0.971	0.158	0.075	0.941	1.003
**Total cholesterol**	0.860	0.487	0.007*	0.769	0.901
**LDL cholesterol**	1.173	0.669	0.005*	1.049	1.312
**HDL cholesterol**	1.163	0.676	0.009*	1.038	1.304
**Triglycerides**	1.128	0.010	0.006*	1.067	1.148
**Creatinine**	0.245	0.322	0.284	0.189	3.206
**eGFR**	0.978	0.108	0.215	0.944	1.013
**Microalbuminuria**	1.003	0.003	0.174	0.999	1.019
**24 h Urinary Sodium**	1.004	0.004	0.303	0.996	1.013
**SBP**	1.203	0.270	0.409	0.775	1.868
**DBP**	0.628	0.427	0.495	0.165	2.385
**PP**	0.737	0.232	0.335	0.397	1.369
**MBP**	1.337	0.837	0.643	0.391	4.564
**CSBP**	1.006	0.382	0.858	0.934	1.072
**Ai 75**	1.021	0.255	0.392	0.972	1.072
**IMT**	1.128	0.002	0.041*	1.031	1.219

* p-value < 0.05.

*BMI* body mass index, *LDL* low-density lipoprotein cholesterol fraction, *HDL* high-density lipoprotein cholesterol fraction, *eGFR* estimated glomerular filtration rate, *SBP* systolic blood pressure, *DBP* diastolic blood pressure, *PP* pulse pressure, *MBP* mean blood pressure, *CSBP* central systolic blood pressure, *Ai 75* augmentation index at 75 beats per minute, *IMT* carotid intima media thickness.

**Figure 3:**
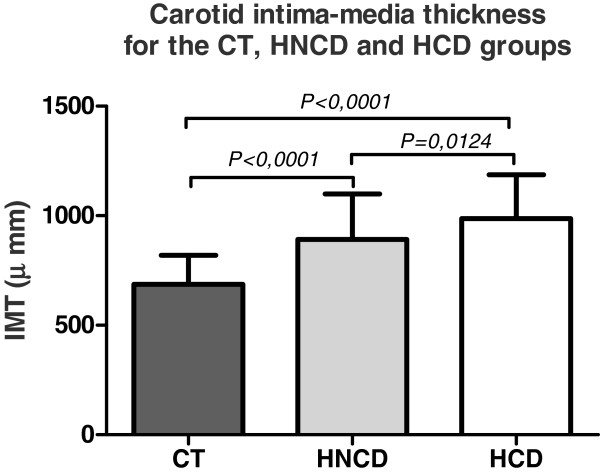
**Evaluation of the carotid intima-media thickness for the CT, HNCD and HCD groups. **IMT = carotid intima-media thickness; CT – controls; HNCD = arterial hypertension without cognitive deficiency; HCD = arterial hypertension with cognitive deficiency.

The results of carotid IMT measurement in the three groups (HCD, HNCD and CT) are shown in Figure 3. The HCD group had a significantly greater IMT compared to the CT group (0.99 ± 0.2 mm versus 0.69 ± 0.1 mm, respectively; p-value < 0.0001). Similarly, there was a statistically significant difference between the HNCD and CT groups (0.89 ± 0.2 mm vs. 0.69 ± 0.1 mm, respectively; p-value < 0.0001). On the other hand, we also observed statistically significant differences for the IMT between the HCD and HNCD groups (0.99 ± 0.2 mm vs. 0.89 ± 0.2 mm, respectively; p-value = 0.0124).

**Figure 4:**
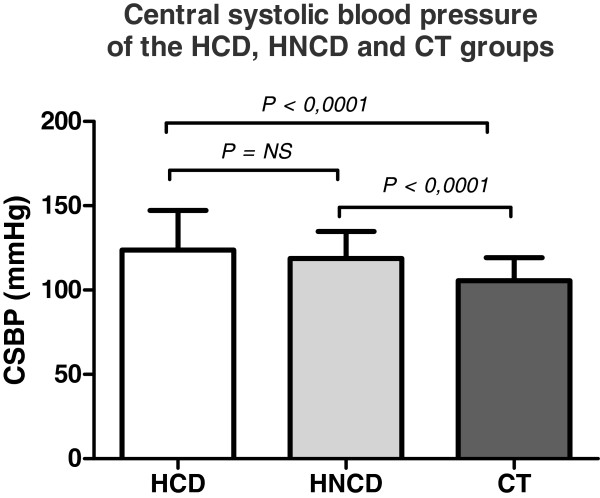
**Central systolic blood pressure for the HCD, HNCD and CT groups. **CSBP = central systolic blood pressure; HCD = arterial hypertension with cognitive deficiency; HNCD = arterial hypertension without cognitive deficiency and CT = controls.

Table 6 shows the CSBP and Augmentation Index (Ai75) corrected for HR for the three groups (HCD, HNCD and CT) and Figure 4 shows the results of the measurement of the central SBP for the three groups (HCD, HNCD and CT). The CSBP was significantly higher for the HCD group compared to the CT group (123.8 ± 3.91 mmHg vs. 105.5 ± 2.0 mmHg, respectively; p-value < 0.0001). Similarly, there was a statistically significant difference between the HNCD group and the CT group (118.5 ± 1.58 mmHg vs. 105.5 ± 2.0 mmHg, respectively; p-value < 0.0001). On the other hand, no significant difference was observed for the CSBP between the HCD and HNCD groups (123.8 ± 3.91 mmHg versus 118.5 ± 1.58 mmHg; p-value = 0.245).

**Table 6:** Central systolic blood pressure and Augmentation Index with correction for a mean heart rate of 75 beats per minute

**Group**		**Mean**	**SD**	**95% CI**
**CT**	**CSBP (mmHg)**	103.6	18.99	80.93 - 90.50
	**Ai 75 (mmHg)**	85.11	15.96	80.48 - 89.75
**HNCD**	**CSBP (mmHg)**	118.8	15.99	15.6 - 121.9
	**Ai 75 (mmHg)**	88.85	9.77	86.93 - 90.77
**HCD**	**CSBP (mmHg)**	120.9	28.56	111.5 -130.3
	**Ai 75 (mmHg)**	89.04	17.15	86.28 - 94.68

*HCD* hypertensive with cognitive deficiency, *HNCD* hypertensive without cognitive deficiency, *CT* controls, *CSBP* central systolic blood pressure, *Ai 75* Augmentation Index at 75 beats per minute.

There were no statistically significant differences in respect to the Augmentation Index (corrected for HR) between the three groups (HCD, HNCD and CT).

## Discussion

The main results of this study show: 1) morphological changes characterized by an increased carotid IMT and functional changes qualified as an increase in central SBP in hypertensive patients with CD; 2) Among the risk factors for the development of CD, the age, HDL cholesterol, LDL cholesterol, triglycerides and increased IMT were significantly associated with a drop in MMSE scores in hypertensive patients.

Several factors have been associated with loss of cognitive function in older adults. According to many studies the educational level is considered to be one of the most important determinants [17,59,60] with less well educated individuals being more likely to have CD. Age has also been linked directly to a loss of cognitive function [61-65]. Other factors have also been mentioned in studies such as being female [66], smoking [67,68], atherosclerosis [69,70], diabetes mellitus [17,71,72], family history of dementia [73] and low-income [17,74].

In this study, the atherosclerotic profile, confirmed by increases in LDL cholesterol, triglycerides and subclinical atherosclerosis characterized by increased carotid IMT were associated with higher ORs for cognitive dysfunction in hypertensive patients independently of the pharmacological antihypertensive treatment and the other risk factors for cardiovascular disease.

Elevation of LDL cholesterol levels increase the risk of cognitive dysfunction by 17% in hypertensive patients in agreement with a few previous reports that showed negative effects of the atherosclerotic lipid profile on the cognitive function in adults [30,75].

On the other hand, epidemiological studies in Rotterdam, Netherlands, demonstrated a significant association between various cardiovascular risk factors including hypertension [76], cerebral white matter lesions and worse performance in neuropsychometric tests [59,60,75,77,78]. Additionally, Ikeda et al. [79], in an analysis of 76 untreated hypertensive patients, 173 treated hypertensive patients and 69 normotensive individuals, showed that the non-treated hypertensive subjects had a higher prevalence of asymptomatic cerebral lacunae at computed tomography compared to normotensive individuals. In this study, the severity and duration of hypertension had a direct correlation with cerebrovascular complications. Studies with large samples showed comparable findings. Liao et al. [79], for example, studied 1920 subjects using magnetic resonance imaging. Hypertension, smoking and age were associated with a higher prevalence of cerebral white matter lesions although the authors did not investigate possible associations between these lesions and CD. Clinical and epidemiological studies also showed positive results in the analysis of this association. Starr et al. [31] in a cross-sectional study of 598 elderly, identified a lower mean MMSE score for individuals with high SBP compared to individuals with normal or low SBPs. Prince et al. [32] performed a case–control study in a clinical trial designed to investigate hypertension treatment in the elderly; a subsample of 50 dementia cases and 223 controls was selected. The study showed that systolic hypertension, without increases in the DBP, was identified as a risk factor for dementia. Launer et al. [80] identified an 8% increase in the risk of CD for each 10 mmHg increase in SBP among 3,735 Japanese Americans. Freidl et al. [81], studying a sample of 1927 healthy elderly in the United States, found among the 16 sociodemographic, environmental and behavioral factors investigated that only age and the presence of hypertension were associated with impaired cognitive performance. Our study also showed that age was significantly associated with a decline in the MMSE scores irrespective of BP, biochemical markers and anthropometric variables.

Scuteri et al. demonstrated the role of vascular health in determining cognitive function in older individuals using multiple regression models and showed that arterial stiffness was a strong predictor of loss of cognitive function independent of age, gender, education and traditional cardiovascular risk factors [82].

Similarly, vascular remodeling detected by estimating the carotid IMT has been linked to increased risk of cardiovascular, coronary and brain events; this represents an important step in carotid plaque formation and progression and is a characteristic marker of atherosclerosis. [83-85]. Of the different pathophysiological explanations, an association between increased IMT and greater pressure variations found in hypertensive patients with vascular remodeling has been described [86]. On the other hand, Forman et al. reported that endothelial-dependent and endothelial-independent vascular responsiveness was correlated with neurocognitive performance among older cardiovascular disease patients, particularly in respect to the attention-executive domain. [87]

With clinical observation, there is a non-consensual understanding that the different forms of hypertension and the results of complications in target organs, related to stiffening of the vascular wall and atherosclerotic plaque formation among other things, are associated to micro- and macro-infarcts of the white substance and is an important causal agent of CD [60,76,88,89]. However, there are few studies in the literature on the association of vascular structural alterations and CD. The results of the current study demonstrate that structural alterations characterized by increased carotid IMT are significantly associated with hypertension in the hypertensive group with CD when compared to hypertensive patients without CD and the CT group and may provide additional information to conventional risk factors for cognitive dysfunction.

There are also few publications in the literature regarding functional hemodynamic changes in hypertensive patients with CD. Our study shows that hypertensive patients, both with and without CD, have significantly higher CSBP compared to the CT group. However, there was no statistically significant difference in the CSBPs of hypertensive patients with and without CD.

There were no statistically significant differences for the Augmentation Index corrected for HR between the three groups (HCD, HNCD and CT).

Several hypotheses may explain the absence of significant differences between hypertensive patients with and without CD. Antihypertensive therapy using renin-angiotensin-aldosterone blockers and calcium channel blockers interferes in the hemodynamics of arterial, central and peripheral BP thus favoring a reduction in the height of the central pressure wave, thereby reducing the Augmentation Index and the central SBP. However, this does not invalidate the important structural finding represented by the main alteration found in the hypertensive group with CD; vascular remodeling characterized by increased carotid IMT.

Potentially confounding effects of antihypertensive therapy on vascular structural and hemodynamic changes may influence cognitive function. In this study the antihypertensive medication did not influence the results (specifically beta-blockers, ACE inhibitors, ARBs and calcium channel blockers).

This study has some limitations: the results are based on a cross-sectional study and so a longitudinal approach is needed to confirm the influence of age on results. Additionally we cannot exclude the influence of genetic factors on the pathophysiology of CD in hypertensive patients. However, new opportunities for structural and functional hemodynamic evaluations are presented for prospective studies on hypertensive patients who have risk factors for CD which may reinforce the importance of achieving the goal in the treatment of hypercholesterolemia in high-risk patients to prevent CD.

## Conclusions

Hypertensive patients with CD have vascular morphological changes characterized by increased carotid IMT, enhanced atherosclerotic lipid profile and impaired hemodynamic functional manifested by elevated central systolic blood pressure.

## Competing interests

The authors declare that they have no competing interests.

## Authors’ contributions

EMD: participated in the study design, clinical assessment of participants, ultrasound examinations, participated in the discussion of the results and helped to write the manuscript. LTG Jr: participated of the clinical assessment of participants, measurement of the central blood pressure and in the discussion of the results.DDM: participated in the collection of clinical data, the blinded measurement of the ultrasound examinations and helped to draft the manuscript. CM: participated in data collection, assessments of peripheral blood pressure and the central blood pressure examination and elaborated the figures. HM Jr: participated of the study design, assessment of the methodology, blinded evaluation of ultrasound examinations and helped with the final discussion. JFVM: participated in the clinical assessment of patients and participated in the discussion of the results. JCYT: participated of the study design, methodological assessment, statistical review, discussion of the results and helped to write the manuscript, corrected the final version in English and submitted the article. All authors read and approved the final manuscript.
